# Assessment of corneal nerve regeneration after axotomy in a compartmentalized microfluidic chip model with automated 3D high resolution live-imaging

**DOI:** 10.3389/fncel.2024.1417653

**Published:** 2024-07-15

**Authors:** Noémie Bonneau, Anaïs Potey, Frédéric Blond, Camille Guerin, Christophe Baudouin, Jean-Michel Peyrin, Françoise Brignole-Baudouin, Annabelle Réaux-Le Goazigo

**Affiliations:** ^1^Sorbonne Université, INSERM, CNRS, IHU FOReSIGHT, Institut de la Vision, Paris, France; ^2^Centre Hospitalier National d’Ophtalmologie des Quinze-Vingts, INSERM-DGOS CIC 1423, IHU FOReSIGHT, Paris, France; ^3^Inserm-DGOS CIC 1423, IHU Foresight, Centre Hospitalier National d’Ophtalmologie des Quinze-Vingts, Paris, France; ^4^Hôpital Ambroise Paré, APHP, Université Versailles-Saint-Quentin-en-Yvelines, Boulogne-Billancourt, France; ^5^UMR8246, Inserm U1130, IBPS, UPMC, Neurosciences Paris Seine, Sorbonne Université, Paris, France; ^6^Faculté de Pharmacie de Paris, Université Paris Cité, Paris, France

**Keywords:** axotomy, corneal neurons, pain, nerve regeneration, microfluidic, compartmentalized culture

## Abstract

**Introduction:**

Damage to the corneal nerves can result in discomfort and chronic pain, profoundly impacting the quality of life of patients. Development of novel *in vitro* method is crucial to better understand corneal nerve regeneration and to find new treatments for the patients. Existing *in vitro* models often overlook the physiology of primary sensory neurons, for which the soma is separated from the nerve endings.

**Methods:**

To overcome this limitation, our novel model combines a compartmentalized microfluidic culture of trigeminal ganglion neurons from adult mice with live–imaging and automated 3D image analysis offering robust way to assess axonal regrowth after axotomy.

**Results:**

Physical axotomy performed by a two-second aspiration led to a reproducible 70% axonal loss and altered the phenotype of the neurons, increasing the number of substance P-positive neurons 72 h post-axotomy. To validate our new model, we investigated axonal regeneration after exposure to pharmacological compounds. We selected various targets known to enhance or inhibit axonal regrowth and analyzed their basal expression in trigeminal ganglion cells by scRNAseq. NGF/GDNF, insulin, and Dooku-1 (Piezo1 antagonist) enhanced regrowth by 81, 74 and 157%, respectively, while Yoda-1 (Piezo1 agonist) had no effect. Furthermore, SARM1-IN-2 (Sarm1 inhibitor) inhibited axonal regrowth, leading to only 6% regrowth after 72 h of exposure (versus 34% regrowth without any compound).

**Discussion:**

Combining compartmentalized trigeminal neuronal culture with advanced imaging and analysis allowed a thorough evaluation of the extent of the axotomy and subsequent axonal regrowth. This innovative approach holds great promise for advancing our understanding of corneal nerve injuries and regeneration and ultimately improving the quality of life for patients suffering from sensory abnormalities, and related conditions.

## 1 Introduction

The cornea, being the most densely innervated tissues in the body (Marfurt et al., 2010), plays a crucial role in maintaining ocular health and function (Al-Aqaba et al., 2019). Corneal nerve fibers originating from the ophthalmic division of the trigeminal ganglion (TG) release multiple neuromediators that maintain corneal epithelial homeostasis and stimulate wound healing once injured. Damage to the corneal nerves, whether due to conditions such as dry eye disease, corneal nerve injury (corneal refractive surgery, diabetic neuropathy, trauma…) can result in chronic corneal neuropathic pain, discomfort, and compromised vision (Belmonte et al., 2017; Guerrero-Moreno et al., 2020). The impact of ocular pain extends beyond physical discomfort, affecting various aspects of daily life, including work and social interactions (Belmonte et al., 2017).

In the last decades, several therapeutic approaches have been proposed to stimulate corneal nerve regeneration and restore corneal nerve function. Among them, recombinant human Nerve Growth Factor (NGF) eye drops, have been the focus of many developments in this field but have only been approved to treat corneal ulcers (Cenegermin Oxervate™) (Ahuja et al., 2020) (Tirassa et al., 2018). Other treatments, such as autologous serum or plasma rich in growth factors (such as Endoret® technology), have shown encouraging results for increasing corneal nerve density, reducing corneal pain and photoallodynia (Aggarwal et al., 2015; Abedi and Hamrah, 2018; Anitua et al., 2022). There is a growing interest in the development of *in vitro* models that can accurately simulate physiological or stimulated corneal nerve regeneration following nerve injury since axonal regrowth is a challenge and a major therapeutic objective.

Over the past several years, microfluidic compartmentalized neuronal cultures that enable the separation and independent study of neuronal somas and axons have emerged as a promising technology to explore neuronal function and plasticity. Despite these advances, there are currently no available data concerning trigeminal nerve regeneration after axotomy using microfluidic compartmentalized chips. Indeed, only one recent study presented compartmentalized models of primary sensory neurons from dorsal root ganglia (DRG) exposed either to inflammatory mediators (mimicking inflammatory peripheral sensitization) or paclitaxel (chemotherapy sensitization) (Giorgi et al., 2023). We recently developed and validated a microfluidic co-culture between mouse trigeminal sensory neurons (responsible for corneal innervation) and primary mouse corneal epithelial cells to evaluate direct toxicity on nerve endings entangled in epithelial cells and indirect toxicity on somas after acute and repeated exposure to benzalkonium chloride, a commonly used preservative in eyedrops (Bonneau et al., 2023). Such microfluidic device allowing a compartmentalization of TG neurons was then considered adequate for investigating trigeminal nerve regeneration after axotomy.

Several *in vitro* axotomy methods in microfluidics have been reported in the literature (Shrirao et al., 2018; Lee et al., 2023): physical methods such as aspiration (Taylor et al., 2005; Nagendran et al., 2017, 2018; Sala-Jarque et al., 2020) or use of a laser (Kim et al., 2009), chemical methods, (saponin, triton, high concentrations of glutamate, etc.) (Kilinc et al., 2011; Li et al., 2012; Samson et al., 2016; Deleglise et al., 2018), and axon compression using pressurized air (Fournier et al., 2015). However, minimizing the variability of the axotomy procedure is still a challenge. In addition, there have been no *in vitro* studies using microfluidic compartmentalized trigeminal neurons to examine the impact of axotomy on cellular changes of calcitonin gene-related peptide (CGRP) and substance P (SP) nociceptive fibers, both expressed in corneal nerve fibers (Jones and Marfurt, 1998), as well as the screening of active compounds on nerve regeneration.

Here, we developed a new approach that combines the microfluidic compartmentalized culture of mouse adult trigeminal neurons with live cell imaging and automated 3D image analysis to investigate the effect of various compounds on axonal regrowth after axotomy. To validate this newly developed axonal regrowth model, we evaluated several compounds with different properties such as nerve growth factor (NGF) combined with glial cell line-derived neurotrophic factor (GDNF) (NGF/GDNF, positive control) (Yang et al., 2021), insulin which was shown to induced epidermal axons regeneration (Guo et al., 2011), an agonist (Yoda-1) and an antagonist of Piezo1 (Dooku-1), a mechanoreceptor that plays a role in axon regrowth (Song et al., 2019; Li et al., 2021), and an inhibitor of the NADase SARM1 (sterile alpha and TIR motif containing 1), an enzyme known to be involved in axon degeneration (Gerdts et al., 2016). By combining compartmentalized neuronal culture with advanced sophisticated live imaging analysis, we confirm that this novel model offers a thorough evaluation of the extent of axotomy and subsequent nerve regrowth. This model could help in identifying compounds that can either enhance or inhibit corneal nerve regrowth, enabling the systematic analysis of various pharmacological compounds.

## 2 Materials and methods

### 2.1 Animals

All experiments were conducted in a specific pathogen-free animal facility. Adult male wild-type C57BL/6 mice, aged to 8 to 12 weeks and weighting between 20 and 25 g, were obtained from Janvier (Le Genest Saint Isle, France). The mice were randomly assigned to cages, with a maximum of five mice, housed in autoclaved 2 L type cages from Allentown equipped with ventilated racks and HEPA filter and enrichment materials. The bedding used was irradiated Aspen bedding (Tapvei, Harjumaa, Estonia). The room was maintained under controlled conditions: a 12:12-h light:dark cycle, with temperature and humidity at the rack level of 22 ± 1°C and 60 ± 10%, respectively. Mice were fed A04 SP-10 irradiated diet (SAFE Diets, Augy, France) and provided autoclaved water in bottle *ad libitum*. A total of 36 animals has been used as trigeminal cell donors in these experiments. All experiments were approved by the Charles Darwin ethics committee (C2E1–05) and conducted in accordance with directive 2010/63/UE of the European Parliament and French Law 2013/118, and the ARVO Statement for the Use of Animals in Ophthalmic and Vision Research.

### 2.2 Reagents

Dulbecco’s Phosphate Buffered Salin (DPBS) (no Ca^2+^, no Mg^2+^; 14,190,144), Neurobasal™-A medium (10888022), horse serum (16050130), and B-27™ (50X; 17,504,044) were purchased from Gibco™ (Thermo Fisher Scientific, Waltham, MA, U.S.). L-Glutamine (G3126), collagenase A (C9891), DNase I (D2650), poly-D-lysine (P6407), laminin (L2020), nerve growth factor (NGF; N6009), glial-derived neurotrophic factor (GDNF; SRP3200), penicillin–streptomycin (P/S 10,000 U/mL; 15,140–122), cytosine β-D-Arabinofuranoside (Ara-C; C1768), fluoro-2′-deoxyuridine (FUDR; 343,333), and insulin (I9278) from Sigma-Aldrich (Saint Quentin Fallavier, France). SARM1-IN-2 (HY-145917), Dooku-1 (HY-126010), and Yoda-1 (HY-18723) were purchased from MedChem Express (CliniSciences, Nanterre, France).

### 2.3 Customized microfluidic chips

**Figure 1:**
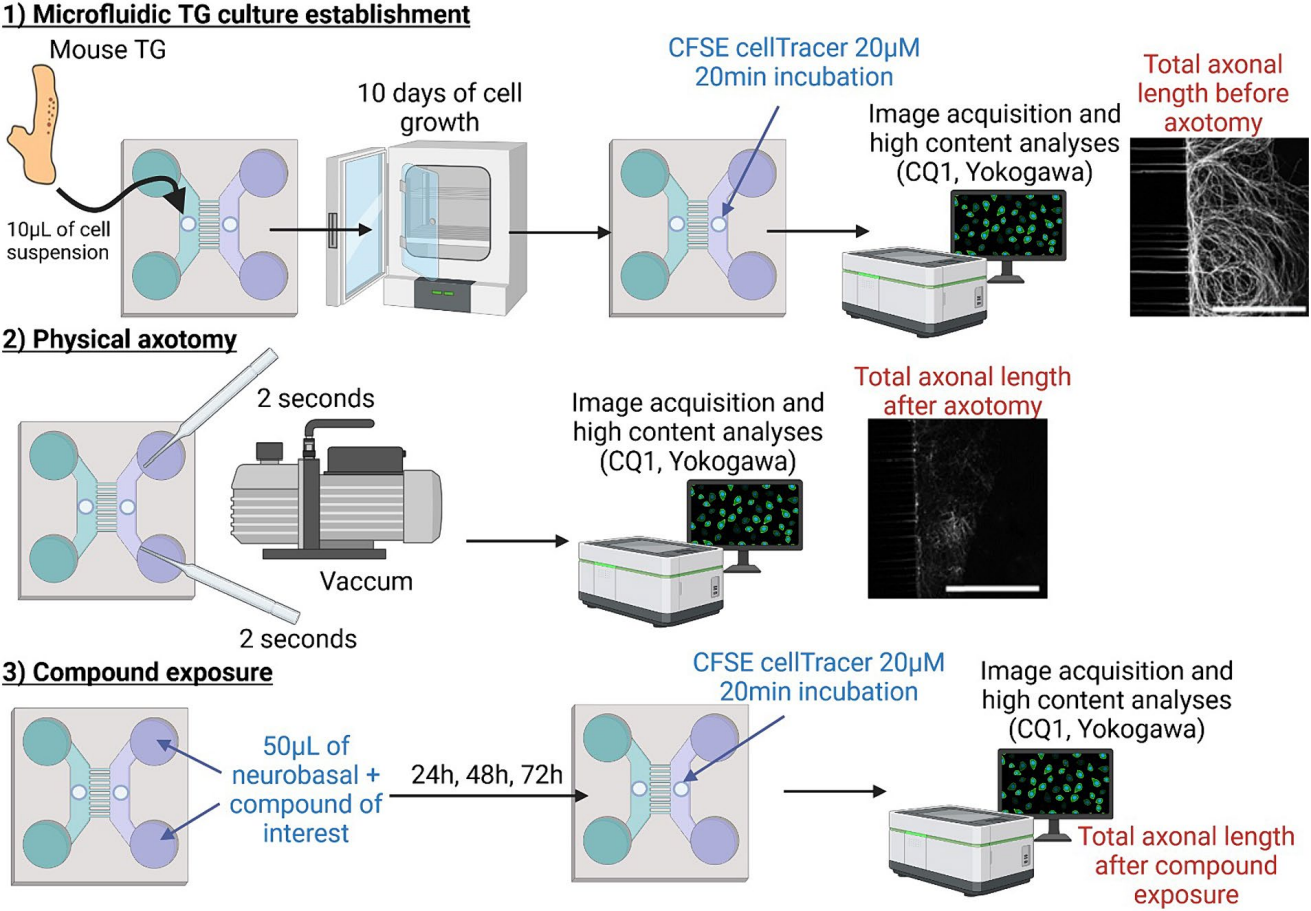
Schematic representation of the axotomy protocol. The first step consisted of seeding dissociated mouse TG cells and establishing the microfluidic device. After 10 days of culture, a physical vacuum-axotomy was performed (step 2). The extent of nerve injury and regeneration was measured using CFSE (alive CellTracer™) before and, and 24, 48, and 72 h after axotomy in the presence of the pharmacological treatment (step 3). Automated 3D image analysis was assessed by using CQ1 associated analysis software. The figure was created using BioRender.com.

Microfluidic chips were designed and manufactured, with the base and curing agent according to the manufacturing protocol of the polydimethylsiloxane (PDMS) chip as previously described (Paul et al., 2011; Courte et al., 2018; Bonneau et al., 2023). Briefly, after PDMS chip demolding, cell seeding inlets were designed by punching 1 mm diameter inlets in each microfluidic chamber near the microchannel structures (Figure 1). After bonding the chip onto a 32-mm diameter cover glass (Menzel-Gläser Thermo Fisher Scientific) and UV-sterilization, a 10 μg/mL poly-L-Lysine coating was applied overnight at 37°C. The next day, after two washes in DPBS, 10 μg/mL laminin coating was applied for 2 h at RT. Upon seeding in the cell culture chambers, the cells were pushed toward the microstructures by microfluidic pressure.

### 2.4 Primary culture of mouse trigeminal ganglion cells

TG cell suspension and seeding in microfluidic PDMS chips was performed as previously described (Bonneau et al., 2023). Briefly, a total of 28 mice were euthanized by CO_2_ inhalation followed by cervical dislocation. Bilateral TG were dissected and placed in Neurobasal™-A Medium. The TG were then enzymatic digested (incubation for 1 h with 2 mg/mL collagenase at 37°C, followed by 10 min in 0.05% Trypsin–EDTA/50 μg/mL DNAse at 37°C) and mechanically dissociated. Following a 10 min centrifugation at 110 g, the cells in suspension were filtered (70 μM; Fisherbrand™ 11,597,522) to eliminate enzymatic residues and debris.

The cell pellet was resuspended at a concentration of 220,000 cells/10 μL quantification performed using a Scepter™ 3.0 handheld cell counter (PHCC30000, with 40 μm sensors, Merck Millipore) in supplemented Neurobasal™-A Medium [5% horse serum, 2% B-27™ (50X), 1% L-glutamine, and 100 U/mL penicillin–streptomycin] (Launay et al., 2016; Vitoux et al., 2020). This seeding concentration helped to reduce animal testing, as it allowed the generation of 4 to 6 chips from the cells of a single mouse. This cell concentrate was introduced into the proximal compartment punch in two stages: two 5-μL injections of the cell suspension with a 15-min interval between the two. The cell suspension was introduced into the seeding inlets, facilitating the flow of cells into the macrochamber where they adhered near the microchannel areas. Following an additional 15-min interval, the microfluidic chips were filled with culture media. To limit flows between the distal and proximal compartment chambers and ensure fluidic compartmentalization, the proximal compartment chambers were over pressurized as follows: 150 μL in each proximal compartment tank (300 μL in total) and 75 μL in each distal compartment tank (150 μL in total). Both microfluidic chip compartments received supplemented neurobasal™-A Medium and NGF/GDNF (neurotrophic factors for promoting axonal growth through the microgrooves) and cytosine-beta-D-arabinofuranoside (Ara-C) /Fluorodeoxyuridine (FUDR) (antimitotic factors to prevent non-neuronal cells from passing through the microgrooves) were introduced to the distal compartment: 100 ng/mL NGF, 100 ng/mL GDNF, 5 μM Ara-C, and 25 μM FUDR. Microfluidic TG cultures were maintained at 37°C in 5% CO_2_ and at 95% humidity. The pressure gradient was renewed every 3 days by removing the media from the compartments and refilling them, as described above, until the axotomy procedure on day 10.

### 2.5 Axotomy procedure and live cell CFSE staining

After 10 days of TG culture, the medium in the distal compartment was removed. Subsequently, 50 μL of 20 μM CellTrace CFSE (Carboxyfluorescein succinimidyl ester; C34554 Invitrogen™) in culture medium per distal compartment inlet was added and the chip incubated for 20 min at 37°C. After one wash, the distal compartment axonal network was directly imaged using a CQ1 Confocal Quantitative image cytometer (Yokogawa, Tokyo, Japan) (see below, Imaging chips with CQ1) using each chip as its own control. The medium in the distal compartment was removed once more and axotomy performed by applying a 2-s aspiration with a Pasteur pipette for each distal compartment inlet, as described by Taylor et al., leading to the formation of an air bubble (Taylor et al., 2005). After one wash with medium, the distal compartment axonal network was again imaged with the CQ1 confocal microscope. Subsequently, compounds of interest (insulin, SARM1-IN-2, Dooku-1, Yoda-1) or control vehicle were randomly added to the distal compartment and renewed every 24 h for a period of 3 days. During each compound renewal, CFSE at 20 μM was once more added to the distal compartment inlet and the chip incubated for 20 min to assess the distal compartment axonal network through CQ1 acquisitions. The procedure is depicted in Figure 1. The percentage of skeleton regrowth was determined by comparing the total axon length in the distal compartment at each timepoint to the length post-axotomy in each device and then normalizing it against the total axonal length before axotomy.

### 2.6 Immunostaining

At the end of the exposure period, the microfluidic chips were rinsed with DPBS and fixed in 4% paraformaldehyde (Pierce™, Thermo Fisher Scientific) for 10 min, followed by three additional washes with 1X DPBS. The cells were permeabilized and non-specific sites saturated by incubation in 0.1% Triton™ X-100 and 3% horse serum in 1X DPBS for 1 h at RT. The chips were then incubated with the primary antibodies overnight at 4°C. If necessary, a pressure gradient was established to enable differential staining between the proximal and distal compartments. After three washes with 1X DPBS, the chips were incubated in secondary antibodies containing DAPI 1:1000 (4′,6-diamidino-2-phenylindole, D9542 – Sigma-Aldrich) for 2 h at RT. The details of the antibody dilutions and references are presented in Table 1. Finally, after three washes, image acquisition and 3D analysis were carried out using the CQ1 (see 2.5).

**Table 1:** Antibodies used for immunohistochemistry.

**Primary Antibodies**		
Anti-beta III tubulin antibody [2G10] - neuronal marker (mouse)	AB78078, Abcam	1:500
Anti-calcitonin gene-related peptide (goat)	1720–9,007, Bio Rad	1:250
Anti-substance P antibody, pain, clone NC1 (rat)	MAB356, Sigma-Aldrich	1:250
Secondary antibodies		
Donkey anti-rat Alexa Fluor™ 488	A21208, ThermoFisher Scientific	1:300
Donkey anti-goat Alexa Fluor™ 594	A11058, ThermoFisher Scientific	1:500
Donkey anti-mouse Alexa Fluor™ 647	A31571, ThermoFisher Scientific	1:500

### 2.7 Imaging chips with CQ1

As previously described (Bonneau et al., 2023), CQ1, a confocal quantitative image cytometer (Yokogawa) was used to acquire images of the chips and perform 3D high-content quantitative analysis using system-provided algorithms CellpathFinder™ Software (Yokogawa). Briefly, a 3D analysis workflow was created for each type of immunostaining for the proximal and distal compartment separately. The algorithm and thresholds were chosen based on the negative control device, ensuring that the selection process is unbiased. Subsequently, the same algorithm was automatically applied to analyze the other devices. This approach prevents any potential bias from the experimenter, who remains blind to the conditions of the devices during the analysis. The automated analysis was conducted on the tile image consisting of the 28 fields. The markers of interest were evaluated in the neurons and/or neurites to obtain the number of positive neurons and the mean length of the axonal skeleton.

### 2.8 Bulk RNAseq analysis of the mouse trigeminal ganglion

Briefly, 8 mice were euthanized by CO_2_ inhalation followed by cervical dislocation. Bilateral TG were dissected and cryopreserved at −80°C until extraction. Total RNA was extracted using a NucleoSpin® RNA XS Macherey-Nagel™ extraction kit (Thermo Fisher Scientific, Waltham, MA, United States), according to the manufacturer’s protocol. Sample purity and quantity were then assessed using a BioAnalyzer 2,100 with the RNA 6000 Nano Kit (Cat# 5067–1,511, Agilent Technologies, Leuven, Belgique). Samples with a minimum RIN of 8 were sent to the iGenSeq core facility at the Institut du Cerveau (Paris, France) for raw read quantification, as previously described (Touhami et al., 2022).

### 2.9 Single cell RNAseq database analysis

In this study, we used the scRNA-seq open data from the trigeminal ganglion cell atlas (https://painseq.shinyapps.io/tg-painseq/), which contains gene expression data from 108,375 mouse trigeminal ganglia nuclei (Yang et al., 2022). The neuronal and non-neuronal cell subpopulations in this atlas were annotated based on the pioneering work of Klein et al. (2015), which employed droplet barcoding for single-cell transcriptomics. We used this atlas to analyze the basal expression of SP, CGRP, growth factors and their receptors, as well as nerve degeneration proteins, by the various subpopulations of mouse TG cells. We further assessed the co-expression of each identified marker with *Tac1* (encoding SP) and *Calca* (encoding CGRP).

### 2.10 Statistical analysis

Statistical analysis was performed using GraphPad Prism 8.0 (GraphPad Software, La Jolla, CA, United States). The results are presented as means ± SD. A Mann–Whitney non-parametric test for comparison between two groups was conducted. For the evaluation of axonal regrowth over time, a two-way ANOVA followed by Tuckey’s correction was performed. Values were considered statistically significant when *p* < 0.05 (*), *p* < 0.01 (**) and *p* < 0.001 (***). The number of replicates (n) is indicated in each figure. Each replicate corresponds to an independent experiment, trigeminal cells coming from different mice.

## 3 Results

### 3.1 Development of a compartmentalized microfluidic chip model for trigeminal nerve axotomy

**Figure 2:**
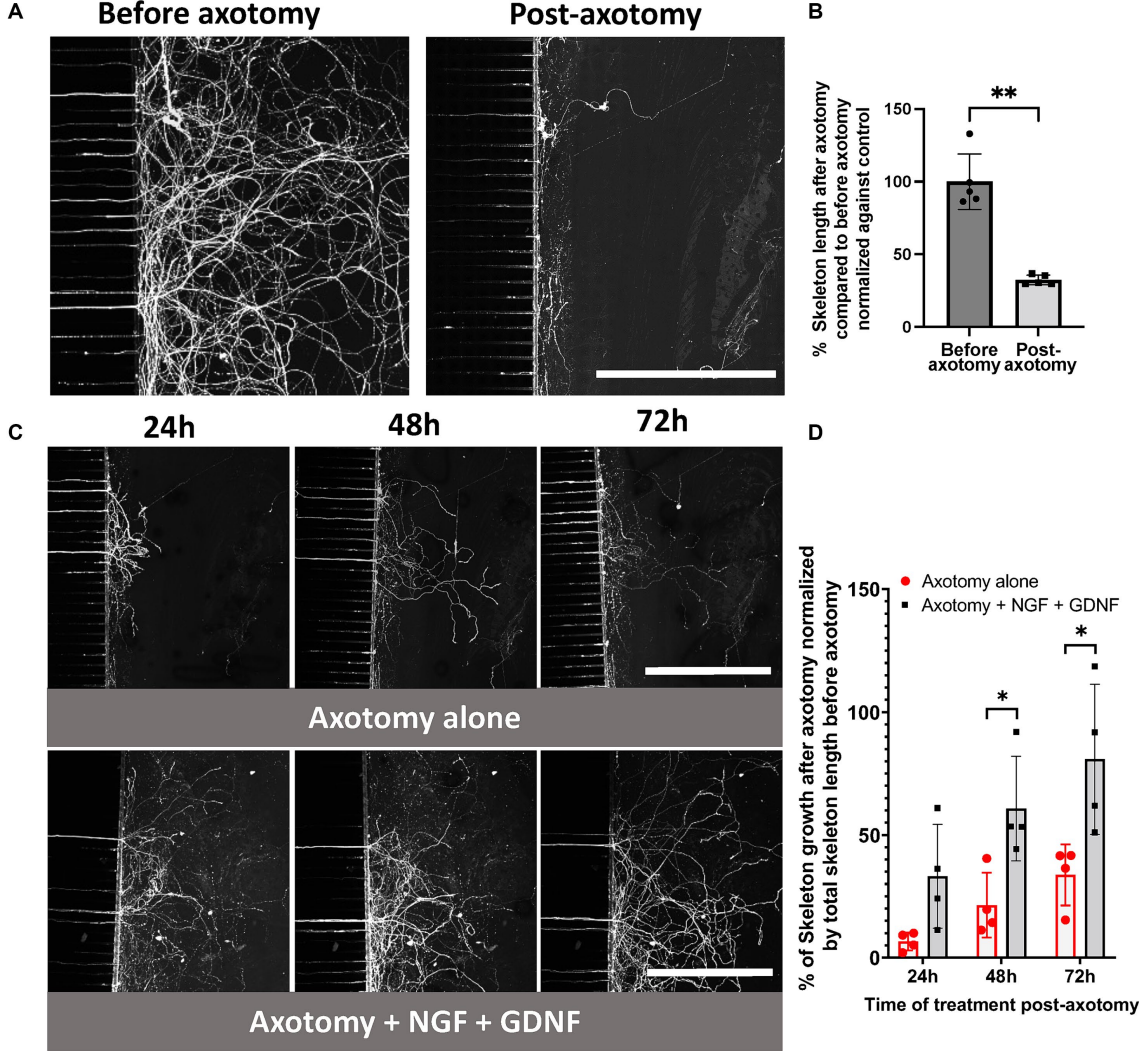
Establishment of a compartmentalized microfluidic axotomy model of primary sensory trigeminal neurons. **(A–C)** CQ1 confocal images and **(B–D)** associated quantification of the length of the axonal skeleton stained with CFSE, before **(A,B)** and after 24, 48, 72 h post-axotomy **(C,D)** in the presence or absence of 100 ng/mL NGF + GDNF. Scale bar = 400 μm. The percentage of skeleton regrowth was determined by comparing the total axonal length in the distal compartment at each timepoint with the length post-axotomy in each device and then normalized against the total axonal length before axotomy. *N* = 4. Mann–Whitney non-parametric test or two-way ANOVA followed by Tuckey’s correction: *p < 0.05; **p < 0.01; ***p < 0.001 compared to control (the significance of treatment effect was determined by comparing to the values to those following axotomy alone at the corresponding time point).

Based on our recent microfluidic neuroepithelial chip model (Bonneau et al., 2023), we developed a microfluidic chip composed of a cellular compartment (proximal compartment) and an axonal compartment containing trigeminal nerve endings (distal compartment) (Figure 1). After 10 days of culture, we exposed the nerve endings to 20 μM CFSE, a cell-permeable molecule, to label the axon skeleton for live imaging. We simulated a peripheral nerve lesion similar to post-surgery or post-injury conditions by performing an axotomy by aspiration using a Pasteur pipette coupled to a suction system for a duration of 2 s (Figure 1). We used CFSE staining of the nerve endings and image acquisition using CQ1 to quantify the length of the axonal network before and after nerve injury, each chip being its own control in our experimental design. Axotomy resulted in the loss of 67.60 ± 2.86% of the axonal network (Figures 2A,B). As highlighted by the low standard deviation, axotomy using this method was highly reproducible between experiments.

Subsequently, spontaneous nerve recovery was monitored at 24, 48, and 72 h using CFSE. The conditions included a baseline control, with no compound (axotomy alone, Figures 2C,D) or NGF at 100 ng/mL (equivalent to 3.77 nM) combined with GDNF at 100 ng/mL (equivalent to 6.62 nM), corresponding to the positive control and reference treatment (Figures 2C,D). Following axotomy, we observed spontaneous axon regrowth of 7, 21, and 34% (relative to the initial network before axotomy) was observed in the distal compartment at 24, 48, and 72 h, respectively.

Exposure to NGF/GDNF improved nerve regeneration, with an increase of 33, 61 and 81% in the distal compartment at 24, 48, and 72 h, respectively (Figures 2C,D). Thus, TG neuronal growth was promoted by this combination of neurotrophic factors.

### 3.2 Phenotypic changes of nociceptive neurons after axotomy

**Figure 3:**
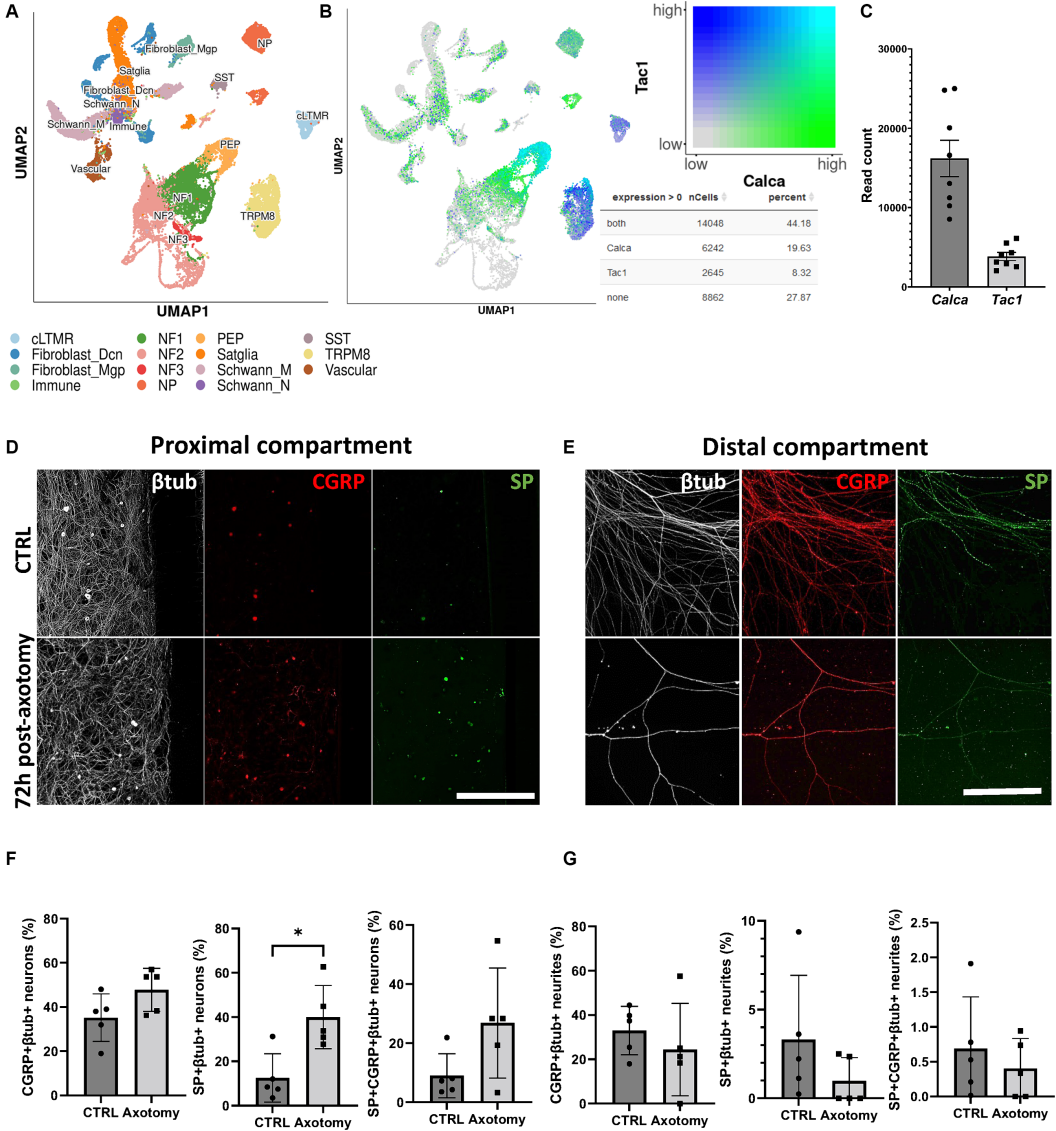
Effect of axotomy on the percentage of trigeminal CGRP^+^ and SP^+^ neurons. **(A)** scRNAseq TG cell subpopulations extracted from database analysis (Yang et al., 2022). **(B)** *Calca* and *Tac1* coexpression extracted from scRNAseq database analysis (Yang et al., 2022). **(C)** *Calca* and *Tac1* read counts in TG obtained by RNAseq analysis. **(D–G)** CGRP and SP immunoreactivity before and at 72 h post-axotomy. **(D,E)** Representative CQ1 confocal images of SP and CGRP immunoreactivity and **(F,G)** quantification of SP and CGRP in (D-F) βtub^+^ neurons in the proximal compartment and **(E–G)** axons in the distal compartment. Scale bar = 200 μm for panel D, scale bar = 300 μm for panel E. N = 5. Mann–Whitney non-parametric test: *p < 0.05, **p < 0.01, compared to the control. vcLTMR, C-fiber low threshold mechanoreceptor (LTMR); PEP, peptidergic nociceptor; TRMP8, TRMP8^+^ cold thermoreceptor; NP, non-peptidergic neurons; NF1, neurofilament^+^ A-LTMR enriched for A-beta-field; NF2, neurofilament^+^ A-LTMR enriched for A-beta-RA and A-beta-field; NF3, neurofilament^+^ LTMR enriched for A-delta; SST, somatostatin-positive pruriceptors; satglia, satellite glia; Schwann_M, myelinating Schwann cells; Schwann_N, non-myelinating Schwann cells; fibroblast_Dcn, DCN^+^ meningeal fibroblasts; fibroblast_Mgp, MGP^+^ meningeal fibroblast; immune, leukocytes; vascular, endothelial cells.

Peripheral sensitization, neurogenic inflammation, and the associated hyperalgesia in response to nerve injury are initiated and maintained, in part, by the actions of CGRP and SP (Iyengar et al., 2017). Indeed, changes in CGRP and SP neuronal content following nerve injury can contribute to sensory abnormalities. However, it is still unknown how the change in SP and CGRP content occurs in trigeminal neurons following nerve axotomy *in vitro*. We addressed this important question by analyzing the proportion and type of cells expressing the nociceptive markers CGRP and SP using scRNAseq open data and investigated how their proportions changed at 72 h post-axotomy. Analysis of the scRNAseq data indicated that 44.18% of TG cells coexpress *Calca* (CGRP gene) and *Tac1* (SP gene). In addition, 19.63% of TG cells exclusively express *Calca* and 8.32% *Tac1* (Figures 3A,B). *Calca* is highly expressed by peptidergic nociceptors (PEP), non-peptidergic nociceptors (NP), and neurofilament^+^ A-low-threshold mechanoreceptors enriched for A-beta-field (NF1). *Tac1* is also highly expressed by PEP nociceptors but is also present in C-fiber low-threshold mechanoreceptor (cLTMR)- and TRPM8-positive fibers (Figures 3A,B). Similarly, we confirmed that *Calca* is more highly expressed than *Tac1* in TG cells by bulk RNAseq analysis with 16189.3 and 3848.1 read counts, respectively. We therefore assessed the impact of nerve axotomy on the levels of SP and CGRP immunoreactivity both in trigeminal neuronal cell bodies and neurites positive for β-tubulin (Figures 3D–G). At the basal level, the number of CGRP^+^ neurons was higher than that of SP^+^ neurons. At 72 h-post axotomy, the percentage of SP^+^ neurons increased from 12.5 ± 7.5% to 39.9 ± 11.0% (*p* < 0.05), whereas the increase in CGRP+ neurons was much less (35.2 ± 7.8% to 47.8 ± 8.4%) (Figures 3D–F). Neurite CGRP and SP levels did not significantly change in the distal compartment (Figures 3E–G).

### 3.3 Pharmacological studies of axonal regrowth

**Figure 4:**
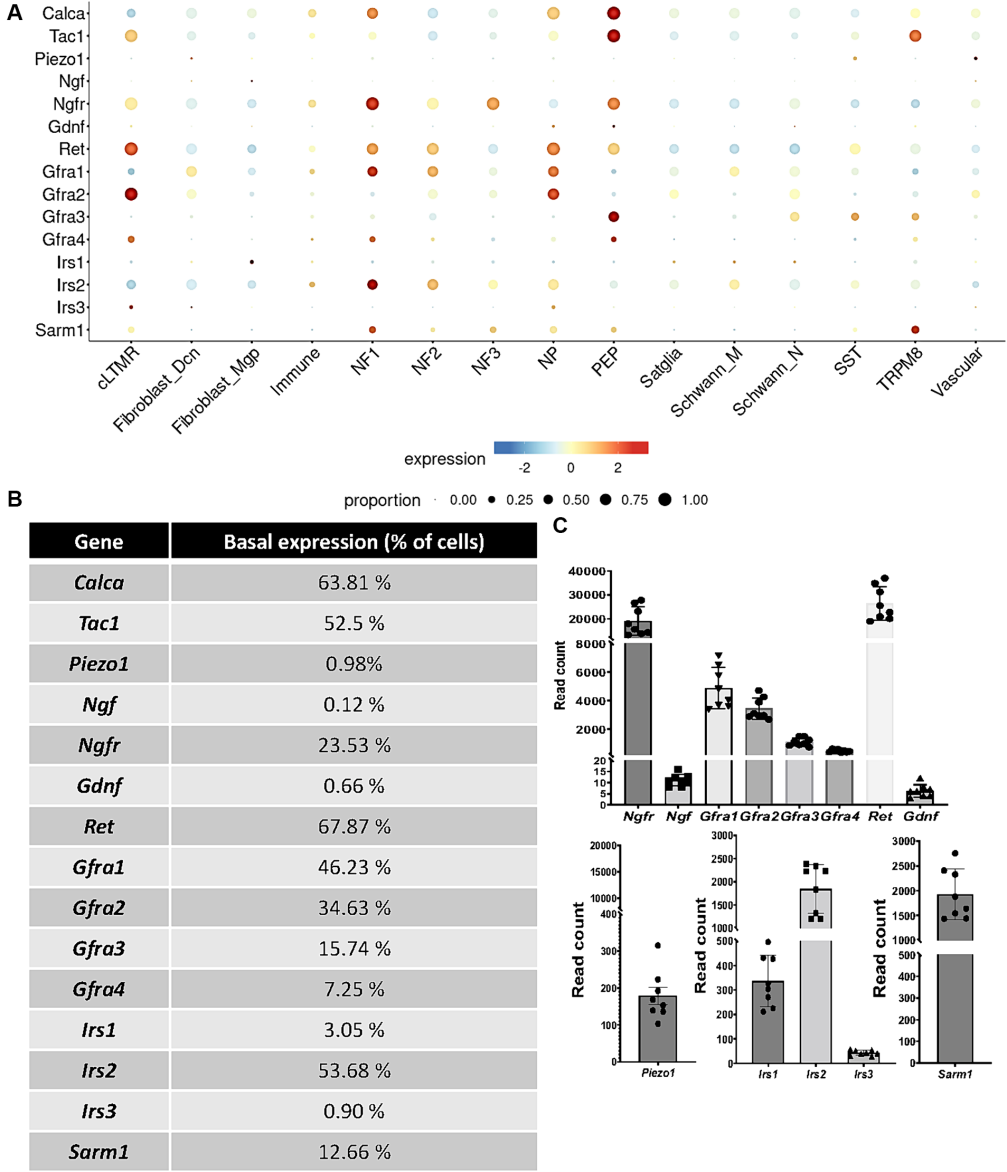
Basal expression of receptors, channels, nociceptors, and growth factors **(A,B)** in TG cell subpopulations extracted from scRNAseq database analysis (Yang et al., 2022). **(C)** read counts of selected targets in TG cells measured by bulk RNAseq analysis *N* = 8 mice. cLTMR, C-fiber low threshold mechanoreceptor (LTMR); PEP, peptidergic nociceptor; TRMP8, TRMP8^+^ cold thermoreceptor; NP, non-peptidergic neurons; NF1, neurofilament^+^ A-LTMR enriched for A-beta-field; NF2, neurofilament^+^ A-LTMR enriched for A-beta-RA and A-beta-field; NF3, neurofilament^+^ LTMR enriched for A-delta; SST, somatostatin-positive pruriceptors; satglia, satellite glia; Schwann_M, myelinating Schwann cells; Schwann_N, non-myelinating Schwann cells; fibroblast_Dcn, DCN^+^ meningeal fibroblasts; fibroblast_Mgp, MGP^+^ meningeal fibroblast; immune, leukocytes; vascular, endothelial cells.

We initiated our study by examining the scRNAseq data obtained from mouse TG cells (Yang et al., 2022) to assess the expression profiles of the pharmacological compounds we previously selected for this study. We first analyzed NGF and GDNF expression along with their respective receptors, as they are two major endogenous neurotrophic factors. Notably, our analysis revealed low expression of both NGF and GDNF within TG cells (0.12 and 0.66%, respectively, of the basal expression in the scRNAseq and < 12 read counts in the RNAseq analysis) (Figures 4A–C). We found the various isoforms of the GDNF receptors to be differently expressed by trigeminal neurons: *Gfra1* is mainly expressed by NF1 and NP (with 46.23% of basal expression, 4,866 read counts in the RNAseq analysis), *Gfra2* mainly by cLTMR and NP (34.63%, 3425.75 read counts), and *Gfra3* mainly by PEP (15.74%, 1094.88 read counts); *Gfra4* is poorly expressed, with only 7.25% of basal expression (463.88 read counts) in cLTMR, NF1, and PEP; *Ret* is highly expressed by cLTMR, NF1, NF2, and PEP (67.87%, 26349.63 read counts). Concerning the NGF receptor, *Ngfr* is expressed in NF1, NF3, and PEP (23.53% of basal expression, 18992.13 read counts) (Figures 4A–C).

We next selected the mechanoreceptor Piezo1, which is poorly expressed by TG cells, with a basal expression of 0.98%, mainly by SST fibers (178.63 read counts). We then investigated the expression of insulin receptors by TG cells. *Irs2* is expressed by NF1 and NF2 trigeminal neurons, with 53.68% of basal expression (1841.38 read counts), whereas *Irs1* is mainly expressed by fibroblasts (3.05%, 336.38 read counts) and *Irs3* by cLTMR fibers (0.90%, 42.75 read counts). Finally, we choose to study the therapeutically targetable metabolic sensor SARM1, which is involved in axonal degeneration (Gerdts et al., 2015; Gilley et al., 2015; Gerdts et al., 2016) and which is expressed by 12.66% of TG cells (1927.5 read counts), mainly by TRPM8 and NF1 fibers, as well as NF2, NF3, NP, and PEP. Thus, scRNAseq basal expression (Figures 4A,B) showed the same tendency as the number of read counts obtained in the TG RNAseq experiment (Figure 4C).

**Figure 5:**
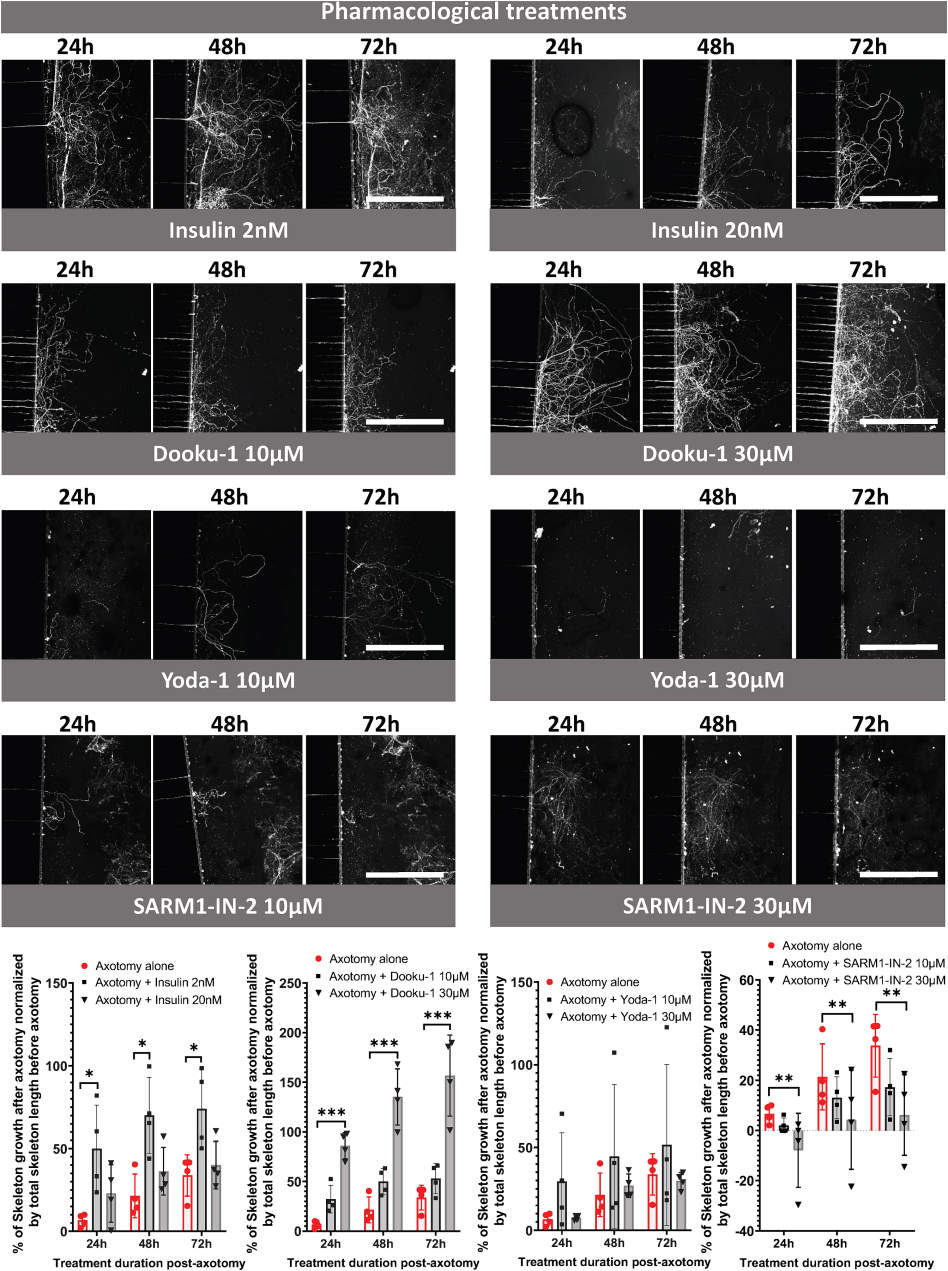
Pharmacological study of trigeminal nerve regeneration in compartmentalized microfluidic chips. CQ1 confocal images and quantification of the length of the axonal skeleton stained with CFSE, at 24, 48, 72 h post-axotomy with or without insulin (2 or 20 nM), Dooku-1 (10 or 30 μM), Yoda-1 (10 or 30 μM) and SARM-IN- 2 (10 or 30 μM). Scale bar = 400 μm. *N* = 4. Two-way ANOVA followed by Tuckey’s correction: *p < 0.05, ***p < 0.001 compared to the control (the significance of the treatment effect was determined by comparing the values to those following axotomy alone at the corresponding time point).

We then carried out a pharmacological study using the following compounds: insulin at 2 nM (physiological dose) and 20 nM, Dooku-1 (Piezo1 antagonist) at 10 μM and 30 μM, Yoda-1 (Piezo1 agonist) at 10 μM and 30 μM, and SARM1-IN-2 (Sarm1 inhibitor) at 10 μM and 30 μM. We assessed the length of the axonal network at 24, 48, and 72 h following pharmacological exposure by CFSE labeling of nerve endings and image acquisition using CQ1 microscope (Figure 5). Insulin at 2 nM and the Piezo1 antagonist (Dooku-1) at 30 μM resulted in a significant increase in the length of the axonal network at 72 h versus axotomy alone: 74 and 154%, respectively (Figure 5). The exposure of trigeminal nerve terminals to 20 nM insulin appeared to be less favorable for axonal regrowth than exposure to 2 nM insulin, resulting in regrowth like that observed without any pharmacological compound. By contrast, neither 10 nor 30 μM Piezo1 agonist (Yoda-1) appeared to influence axonal regeneration. Finally, exposure to the SARM1 inhibitor (SARM1-IN) at 30 μM reduced axonal regeneration at all time points analyzed (Figure 5), leading to regrowth of only 6% after 72 h of exposure (versus 34% regrowth without any compound).

## 4 Discussion

There is a growing interest in the development of *in vitro* models that can accurately simulate physiological or stimulated corneal nerve regeneration following nerve injury since axonal regrowth is a challenge and a major therapeutic objective. Currently, no medical treatment is routinely given in the clinic following corneal nerve injury to improve regeneration of the damaged nerves and restore functional nerve abnormalities. In this context, we first developed a compartmentalized microfluidic trigeminal neuronal culture in which physical nerve axotomy was performed by aspiration to mimic peripheral nerve lesion, such as LASIK or corneal abrasion. This aspiration method was adapted from the works of Taylor et al. (2005) and Nagendran et al. (2017, 2018). To improve the reproducibility and precision of the axotomy, we used a Pasteur pipette, which is finer than a pipette tip, following the complete removal of culture medium. A 2-s vacuum aspiration was performed in each inlet of the distal compartment, significantly reducing vacuuming time from 1 to 2 min (Nagendran et al., 2018). We then combined this homemade axotomy model, live cell imaging, and automated 3D high resolution image quantification methods (Bonneau et al., 2023) of the trigeminal axonal network to evaluate the length of the axonal skeleton before and after axotomy. Notably, the main difference between the commercially available XonaChip® used by Taylor’s laboratory and the homemade PDMS chip used in our studies is the addition of a seeding punch. This feature enhances TG neuron seeding in the proximal compartment, near the microchannels, and facilitates axon crossing into the distal compartment (Bonneau et al., 2023). This methodological design allows for each device to serve as its own control, facilitating reproducible measurements of the axonal network both before and after axotomy, as well as evaluating the effects of drug exposure on axonal regeneration. This new analysis method thus refines previous systematic testing of the neuroregenerative potential of pharmacological compounds and also help to reduce, refine the use of animals, in accordance with the requirements of the 3Rs in research.

### 4.1 Phenotypic changes of SP and CGRP nociceptors following axotomy

Cell culture models allow a high degree of control and monitoring, enabling detailed neuronal responses to be measured. Therefore, compared to monolayer culture studies, our microfluidic device allows to separately analyze the proximal compartment and distal compartment, i.e., neuronal cell body from nerve endings, reflecting distant effects of axotomy on trigeminal neuronal cells. In this context, we reported precise changes in SP and CGRP expression by the cell bodies and nerve fibers following axotomy and the evaluation of axonal regrowth. SP and CGRP are produced in a subset of polymodal peptidergic nociceptors expressed by trigeminal sensory neurons and corneal nerve (Jones and Marfurt, 1998; He and Bazan, 2016). Both are major neuropeptides, involved in corneal pain processing and inducing epithelial cell proliferation, migration, and facilitating corneal wound healing (Garcia-Hirschfeld et al., 1994; Puri et al., 2022). Three days-post axotomy, we found that axotomy only increases the number of SP^+^ cell bodies and not the number of CGRP^+^ neuron subpopulation. Moreover, axotomy did not induce any significant changes (only a trend toward a decrease) in the proportion of SP^+^ and CGRP^+^ fibers. The increased expression of SP in TG neurons in our *in vitro* model is consistent with observations from *in vivo* studies. Indeed, we recently reported higher expression of *Tac1* gene, encoding SP, in mouse TG (Fakih et al., 2021), but no change in the expression of the *Calca* gene, encoding CGRP, in a corneal pain model induced by persistent dry eye disease (data not shown). It has been described that peripheral nerve injury induces neuropeptide biosynthesis and *de novo* expression of SP in neurons that are typically negative for SP (Neumann et al., 1996; Ruscheweyh et al., 2007; Byun et al., 2020). Furthermore, Noguchi et al. reported that peripheral nerve axotomy dramatically increases the expression of the SP precursor, preprotachykinin mRNA, and SP levels in DRG neurons (Noguchi et al., 1994; Weissner et al., 2006). In another non-clinical model of corneal damage, Byun et al. (2020) demonstrated a significant increase in the proportion and mean cell size of corneal trigeminal neurons with SP immunoreactivity in animals treated with 0.1% benzalkonium chloride (BAK). The authors suggested that BAK-induced ocular surface damage led to *de novo* SP expression in corneal trigeminal neurons, which normally do not express SP. Based on these studies, the increased number of SP+ neurons observed in our experimental conditions likely results from *de novo* SP synthesis. Further quantitative molecular analyses are needed to confirm this *de novo* synthesis. Overall, these data highlight the robustness of our *in vitro* model for the detection of cellular changes following trigeminal nerve axotomy.

### 4.2 scRNAseq datasets and target identification for nerve regeneration

Although nerves are known to naturally partially regenerate over time, achieving complete recovery is challenging (Navarro et al., 2007). Analyses of scRNAseq datasets are increasingly used to profile cellular composition at high resolution, leading to the discovery of potential therapeutic targets and offering an unprecedented opportunity to study specific biological questions (Hsieh et al., 2023). Here, we used a scRNAseq TG dataset (Yang et al., 2022) to select pharmacological targets and their potential interest in nerve regeneration. The selected targets showed varying levels of basal expression, ranging from high expression, such as that of *Irs2*, present in 53.68% of TG cells, to less highly expressed targets, such as *Piezo1*, which is expressed in only 0.98%. Using this broad spectrum of targets, we aimed to validate the synergy between scRNAseq data base analysis for target identification and the evaluation of axonal regeneration in a novel model of axotomy developed in a microfluidic chip.

### 4.3 Neurotrophins and axonal nerve regeneration

Nerve growth factors, in particular neurotrophins, are released in response to nerve injury and play a role in promoting nerve regeneration (Yang et al., 2021). Among such factors, NGF has been considerably studied, as it is expressed and released by corneal epithelium and keratocytes (Qi et al., 2008). This mediator, together with GNDF, represents the main actors of the interplay between corneal nerve fibers and corneal epithelial cells for the normal healing of the cornea. We observed that the exposure of injured nerve endings to NGF combined with GDNF improved axonal regrowth. These observations are consistent with those of an *in vivo* study in rabbits that underwent corneal refractive surgery (Gong et al., 2021). Indeed, NGF-containing eyedrops (200 μg/mL) improved the recovery of corneal nerve density compared to hycosan and physiological saline treatment at one and three months after the surgery. Moreover, recombinant human NGF (rhNGF) eye drops, such as Cenegermin (Oxervate™), have already been formulated and approved in the Unites States for the treatment of corneal ulcers (Ahuja et al., 2020), although they have not yet been approved in the European Union. Moreover, patients with neurotrophic keratopathy treated for at least 4 weeks with rhNGF, showed both a significant increase in corneal nerve densities and in corneal sensation (Balbuena-Pareja et al., 2023). Similarly, the topical administration of enriched plasma containing several growth factors such as in Endoret® technology, shows promise for the treatment of corneal nerve damage (Aggarwal et al., 2015; Abedi and Hamrah, 2018; Agirregoikoa et al., 2022; Anitua et al., 2022; Carreno-Galeano et al., 2023). Finally, autologous serum tears (AST), that contains epidermal growth factor (EGF), NGF, insulin-like growth factor (IGF-1), vitamins, produces improvement in ocular symptoms and morphological abnormalities of corneal nerves in DED patients and patients with neuropathic corneal pain (Aggarwal et al., 2015; Hamrah et al., 2017; Carreno-Galeano et al., 2023).

### 4.4 Piezo 1 and nerve regeneration

Piezo1 is a mechanosensitive ion channel expressed in mouse and human trigeminal sensory neurons and cornea (Roh et al., 2020; Zhu et al., 2021; Mikhailov et al., 2022). Our data demonstrated that Dooku-1, a Piezo1 receptor antagonist, promotes trigeminal nerve growth in a dose-dependent manner that surpassed that of NGF associated with GDNF. Conversely, activation of the Piezo1 channel with the compound Yoda-1 did not modify axonal regrowth at 10 or 30 μM relative to spontaneous axonal regeneration.

The axonal regrowth induced by Piezo1 inhibition is in accordance with previous studies. Indeed, when studying axon regeneration in *Drosophila melanogaster*, in embryonic rat hippocampal neurons (in culture), and in adult mouse DRG neurons, Piezo1 takes on an inhibitory role (Song et al., 2019; Li et al., 2021). The inhibitory effect was proposed to be due by the activation of the ataxia telangiectasia and Rad3-related (Atr)-checkpoint kinase-1 (Chek1) pathways (Li et al., 2021) known to non-canonically inhibit axon regrowth (For review (Miles et al., 2023)). In Drosophila, GCaMP6f imaging revealed increased local Ca^2+^ influxes at the axon tips in wild-type flies compared to Piezo-null flies during the axon regeneration period. This influx occurs before the obvious formation of filopodia in growth cones, suggesting that Piezo1 functions locally to inhibit regeneration (Song et al., 2019). In microfluidic devices, embryonic rat hippocampal neurons were subjected to shear stress to induce injury. When the injured axons were locally treated with Yoda1, a Piezo1 agonist, axon regeneration was significantly reduced, indicating that Piezo1 plays an inhibitory role in axon regrowth following injury (Song et al., 2019). Finally, Velasco-Estevez et al. (2020) demonstrated by using an *ex vivo* murine-derived organotypic cerebellar slice culture model and *in vivo* by co-administering it with lysophosphatidylcholine (LPC), into the cerebral cortex of adult mice, that GsMTx4 (an antagonist of mechanosensitive cation channels such as Piezo1) prevented both demyelination and neuronal damage. Our model is, to our knowledge, the first to report an interest of using Dooku-1, a Piezo 1 receptor antagonist, for neuronal regeneration underscoring the importance of this Piezo1 mechanoreceptor in nerve growth. The precise mechanisms triggered by Dooku-1 warrants further investigation.

Despite the low expression of the Piezo1 receptor in TG cells, the significant effect of exogenous Dooku on axonal regrowth can be attributed to several factors. Firstly, Piezo1 receptors, although expressed in small levels, are highly sensitive mechanotransducers that play a critical role in cellular responses to mechanical stimuli. The inhibition of Piezo1 receptors can initiate downstream signaling cascades that significantly impact axonal regeneration, such as the Atr-Chek1 pathway (Li et al., 2021). Secondly, Dooku may influence axonal regrowth through secondary mechanisms or interactions with other signaling pathways that are modulated by Piezo1. Further studies are needed to elucidate these pathways and fully understand the complex dynamics at play.

### 4.5 Insulin and nerve regrowth

Diabetic neuropathy can manifest in the cornea as a reduction in nerve density, altered nerve morphology, and impaired corneal sensitivity (Markoulli et al., 2018). This condition, often referred as diabetic corneal neuropathy, can lead to symptoms such as discomfort or a foreign body sensation due to dryness or for some patients more by sensory deficits rather than pain. Insulin, the endogenous hormone that regulates glycemia, is known to improve corneal re-epithelialization (Wang et al., 2017; Diaz-Valle et al., 2022) and was shown to trigger epidermal axons regeneration in a mouse model of diabetic neuropathy (Guo et al., 2011). Although insulin receptors are expressed in the nervous system (Lee et al., 2016), the specific impact of insulin on trigeminal nerve regeneration is not known. Here, we found that an insulin concentration of 2 nM, considered to be a physiological concentration, showed greater efficacy in promoting nerve regrowth than a concentration that was 10 times higher. This observation suggests that excessive concentrations of insulin may have detrimental effects on the axonal trigeminal network. Such observations are in line with results obtained in another model. Indeed, in a monolayer culture of DRG neurons, low (nM range) concentrations of insulin promoted axonal growth, whereas higher concentrations (μM range) inhibited it (Lazar et al., 2020).

### 4.6 Inhibition of SARM1 deleterious for axonal regrowth

The NADase SARM1 is primarily recognized as a metabolic sensor that facilitates axonal degeneration (Gerdts et al., 2015; Gilley et al., 2015; Gerdts et al., 2016). Liu et al. explored SARM1 inhibition’s neuroprotective potential across three ocular conditions, yielding diverse outcomes. In optic nerve injury models, targeting SARM1 via intravitreal antisense oligonucleotide or SARM1^−/−^ mice offered axonal protection but not soma preservation. Conversely, in a silicone oil-induced ocular hypertension model, SARM1^−/−^ mice or SARM1-targeting antisense oligonucleotide injections demonstrated neuroprotection for both retinal ganglion cell somas and axons. However, in an experimental autoimmune encephalomyelitis/optic neuritis model, neither SARM1 inhibition strategy improved retinal ganglion cell survival (Liu et al., 2023). Here, we showed that SARM1 inhibitor reduced axonal regeneration of trigeminal nerve fibers, specifically at high concentration (30 μM). These results underscore the complex mechanism of the influence of SARM1 on axonal regeneration, suggesting the need for further in-depth exploration in a context of corneal nerve damage.

## 5 Conclusion

In conclusion, the innovative axotomy microfluidic chip offers a novel approach for assessing peripheral nerve regeneration. It facilitates the direct exposure of nerve endings to pharmacological compounds while preserving nerve physiology. This new methodology not only paves the way for the systematic and robust analysis of the regenerative capacity of compounds for trigeminal nerve fibers but also extends its applicability to other physically altered peripheral fibers. By including high-content quantitative analysis, this model is the first, to our knowledge, to precisely monitor the molecular and cellular responses induced by nerve axotomy. In addition, the potential of this methodology lies in its ability to screen and identify new drug candidates for corneal nerve regeneration.

## Data availability statement

The raw data supporting the conclusions of this article will be made available by the authors, without undue reservation.

## Ethics statement

The animal study was approved by the Charles Darwin ethics committee (C2E1–05). The study was conducted in accordance with the local legislation and institutional requirements.

## Author contributions

NB: Conceptualization, Writing – original draft, Writing – review & editing, Data curation, Methodology. AP: Methodology, Software, Writing – review & editing. FB: Data curation, Methodology, Writing – review & editing. CG: Funding acquisition, Writing – review & editing. CB: Funding acquisition, Writing – original draft, Writing – review & editing. J-MP: Conceptualization, Methodology, Writing – review & editing. FB-B: Conceptualization, Data curation, Funding acquisition, Supervision, Validation, Writing – original draft, Writing – review & editing. AR-LG: Conceptualization, Data curation, Funding acquisition, Supervision, Validation, Writing – original draft, Writing – review & editing.
